# Nervous Headache, &c.

**Published:** 1843-11

**Authors:** 


*Nervous Headache, &c.*—A physician of Marseilles has found headaches of a kind dependent on nervous disturbance, obstinate tic-douloureux, &c., curable by the application of liquor ammoniæ (*l'ammoniaque depuis le ving-cinquieme degré jusqu' au trente-deuxieme*), on a dossil of lint, to the alveolar border of the palate. The solution is to be retained in contact with the mucous membrane immediately within the teeth, until an abundant effusion of tears is excited, when the exacerbation of pain will suddenly cease. This remedy proves more efficient against tic douloureaux attacking the frontal and facial than the occipital nerves; but it has been successful in several authenticated instances in which the latter have been the seat of pain.—*Ibid.*

